# Effect of Increased Flexor Hallucis Longus Muscle Activity on Ground Reaction Force during Landing

**DOI:** 10.3390/life11070630

**Published:** 2021-06-29

**Authors:** Kosuke Oku, Daisuke Kimura, Tomotaka Ito, Akiyoshi Matsugi, Tatsuya Sugioka, Yusuke Kobayashi, Hayato Satake, Tsukasa Kumai

**Affiliations:** 1 Department of Rehabilitation, Kawasaki University of Medical Welfare, Kurashiki, Okayama 701-0193, Japan; kimura.d@mw.kawasaki-m.ac.jp (D.K.); s-ito@mw.kawasaki-m.ac.jp (T.I.); 2 Faculty of Rehabilitation, Shijonawate Gakuen University, Daitou, Osaka 574-0011, Japan; a-matsugi@reha.shijonawate-gakuen.ac.jp; 3 Division of Rehabilitation, Hanna Central Hospital, Ikoma, Nara 630-0243, Japan; ta930331@yahoo.co.jp (T.S.); yusukekobaysi@yahoo.co.jp (Y.K.); 27hyatt@gmail.com (H.S.); 4 Faculty of Sport Sciences, Waseda University, Tokorozawa, Saitama 359-1192, Japan; kumakumat@waseda.jp

**Keywords:** big toe, muscle contraction, biomechanics, surface electromyography

## Abstract

Repeated high-impact ground forces can lead to injury and decreased performance. While increasing flexor hallucis longus (FHL) muscle activity is known to increase stiffness and elasticity, it is unknown if this also decreases ground reaction forces by shock absorption during landing. This study aimed to determine whether increasing FHL muscle activity affects ground reaction force during landing in healthy subjects. Eight subjects performed single-leg steps onto a force platform for five trials, with and without flexion of the metatarsophalangeal (MTP) joint at the moment of landing. Integrated surface electromyography (sEMG) of the FHL and medial gastrocnemius (MG) and ground reaction forces (GRFs) were measured. sEMG and GRF during the 50 ms before and 100 ms following initial ground contact were analyzed and compared. Flexion of the MTP joint condition significantly decreased the vertical and mediolateral force peaks of GRF, and FHL muscle activity increased. Flexion of the MTP joint at the moment of landing reduces GRF in healthy subjects through force dissipation in the foot, by increased FHL muscle activity. The results suggest that this may contribute to injury prevention by reducing the impact force through flexing the MTP joint at the moment of landing.

## 1. Introduction

Injury during sports commonly occurs immediately after initial ground contact during landing activities, and the mechanism includes elevated repetitive impact forces [1]. When the impact forces become higher, muscles or joints cannot adequately dissipate the stresses, and there is a potential for sports injury [2]. Recent studies on injuries during sports have reported that the repetition of impact force might lead to increased injury risks [3,4]. 

When analyzing the impact forces, one should study the internal structure forces for both the impact and active parts of ground contact [5]. Biomechanics studies for sports injury prevention have sought to understand and predict the potential for injury by evaluating the ground reaction force (GRF) [6]. Increases in GRF are assumed to reflect increases in impact force to the internal structure [7], even though the GRF is not necessarily reflective of the actual loading [8,9] on joints, muscles, or bones that causes overuse injury to these internal structures [5]. GRF is related to repetitive impact load accumulation, and overuse injury in the lower extremities [10,11]. Understanding the technique also has important clinical and technological implications for sports injury prevention.

A strong longitudinal arch offers substantial passive resistance to these loads [12]. A recent study reported the static contribution of the ligaments and the dynamic contribution of foot muscles in supporting the longitudinal arch [13]. The activity of the extrinsic foot muscle tendons clearly illustrates their ability to provide dynamic support and control of both the longitudinal and transverse components of the foot dome [14]. Several functions have been attributed to the flexor hallucis longus (FHL), including flexion of the metatarsophalangeal (MTP) joint and maintenance of the medial longitudinal arch of the foot [15,16]. It is unknown whether increasing FHL muscle activity when landing also alters the GRFs.

Thus, FHL muscle activity may manipulate the GRF on landing by enhancing the longitudinal arch of the foot. Therefore, our hypothesis was that increased FHL muscle activity would decrease the impact forces, compared to a natural landing. The purpose of this study was to determine if landing with increased FHL muscle activity decreased impact forces, compared to the natural conditions.

## 2. Materials and Methods

### 2.1. Subjects

Eight male individuals (age 22.3 ± 0.97 years, height 175 ± 1.95 cm, body weight 68.1 ± 4.41 kg), who were physical education students, volunteered to participate. The inclusion criteria were men aged 19 to 24 years; each subject had participated in sports at least three times per month and was active. Subjects with a history of serious lower-extremity injuries were excluded. The right (dominant) leg of each subject was assessed using a previously validated questionnaire [17]. The subjects provided informed written consent prior to their participation, and the study was approved by the Kawasaki University of Medical Welfare Ethics Committee (approval number: 20-065). The study was conducted at the Kawasaki University of Medical Welfare, Japan.

### 2.2. Instrumentation

The landing tasks consisted of stepping off a 30-cm box onto a landing force platform (OR6-6-2000 AMTI, Watertown, MA, USA), sampling at 1000 Hz, to collect the GRF data. A wireless surface electromyography (sEMG) system (WEB-1000, Nihon Koden, Tokyo, Japan) was used to collect the sEMG data from the FHL and medial gastrocnemius (MG) of the right leg at 1000 Hz.

**Figure 1:**
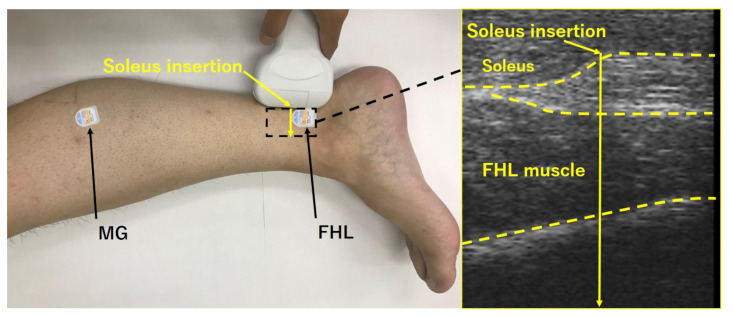
Diagram of surface electrode placement. Flexor hallucis longus (FHL) electrode placement is shown. Diagram of surface electrode placement, adapted from [16]. The insertion of the soleus and the muscle-tendon junction of the FHL was defined by ultrasonography (Miruco, Sigmax, Tokyo, Japan), and surface electromyography electrodes were placed between these anatomical landmarks. MG, medial gastrocnemius.

The skin of the electrode attachment sites was shaved, gently abraded with sandpaper, and cleaned with an alcohol swab before the application of the electrodes. The electrode sites for MG were placed according to the recent guidelines [18]. The electrode was placed over the FHL muscle belly on the medial side of the leg, between the soleus insertion and the FHL muscle-tendon junction, where only the FHL muscle belly lies [19] (Figure 1). Moreover, the proper placement of the electrodes over the FHL was ensured by ultrasonography [16]. The signal quality was confirmed by ultrasound to dissociate from the activation amplitude FHL and MG during MTP flexion. Another factor that might have affected FHL sEMG data was the muscle belly displacement relative to the sEMG electrodes. It might be expected that the FHL contraction shows fascicle length changes, and would alter the relationship between the surface electrodes and the active portion of the muscle, as compared to landing without muscle contraction. However, a recent study found that the range of FHL fascicle length change was relatively small [20]. The strength of the FHL muscle activity signal was confirmed during isolated big-toe plantar flexion. The data were transferred to AD Instruments (PL3508 PowerLab System, Bella Vista, New South Wales, Australia), recorded using LabChart software (AD Instruments, Bella Vista, New South Wales, Australia), and converted into a digital form.

### 2.3. Landing Protocol

**Figure 2:**
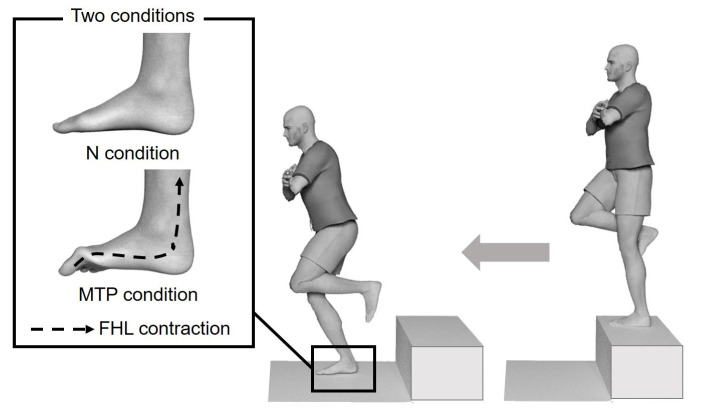
Landing technique.

The subjects stood barefoot on the 30-cm high platform in front of the force plate, with the test leg relaxed and non-weight-bearing. The subjects were instructed to fold their arms across their chest and step off the box using the contralateral leg, without jumping up, and to land in the center of the force plate (Figure 2) [21] under two conditions (Figure 2): the natural condition (N condition), where subjects were instructed to land as they would normally; and the MTP joint flexed condition (MTP condition), where subjects were instructed to “flex the MTP joint when you land.”

Subjects were permitted a period of practice of the technique prior to testing, where they performed five trials on the force plate. All subjects performed both the N and MTP conditions, and were randomly assigned to start with either condition, to eliminate the influence of learning from the results. The subjects were assigned to two groups using a randomized computer-generated sequence. Allocation concealment was performed using a sequentially numbered list, which was used to assign a blinded evaluator to each corresponding participant. The researcher that conducted the allocation was not involved with the study, to ensure effective blinding of the evaluators.

The subject landed on the test leg on the force platform. The subject stood on the support surface with the test leg relaxed and non-weight bearing. The subject landed on the test leg on the force platform. The test was performed under two conditions: the natural condition (N condition), where subjects were instructed to land as they would normally; the metatarsophalangeal (MTP) joint flexed condition (MTP condition), where subjects were instructed to “flex the MTP joint when you land.”

### 2.4. Electromyography and Ground Reaction Force Signal Processing

The sEMG and GRF data were analyzed using LabChart software (ADInstruments, Bella Vista, New South Wales, Australia). 

Ground contact was determined from vertical GRF tracings. sEMG and GRF data, during the 50 ms before and 100 ms following the initial ground contact, were extracted. A literature search indicated that the bandpass filtering generally involved a band-pass filter between 20 and 500 Hz [22] To remove artifacts during landing, the raw sEMG signals were filtered through a band-pass filter between 30 and 500 Hz [23,24]. The sEMG data were then full-wave rectified and averaged within a participant and task. The sEMG values of each subphase were normalized to the peak sEMG during landing from the maximum voluntary contraction (MVC). After the landing protocol was recorded, selected manual muscle testing (MMT) was performed, to permit normalization of the sEMG data. Two maximum isometric voluntary contractions were elicited using MMT techniques in each test position, during which a 3 s recording of the sEMG was obtained [25]. Prior to data analysis, all values of GRF were normalized with respect to each subject’s body weight (BW) in kg to eliminate intergroup differences based on the individual subjects’ BW. From this point on, all parameters of the GRF were expressed as a percentage of BW (%). Magnitudes of peak vertical (GRFv), lateral (GRFl), and medial (GRFm) forces were identified for each jump, and individual and group means were subsequently calculated. Additionally, sEMG data were averaged following impact for each participant, and group means were calculated.

### 2.5. Sample Size

The sample size was determined, based on the assumed percent reduction of GRF for the MTP joint flexed condition, using G*Power 3.1 software (Dusseldorf University, Dusseldorf, Nordrhein-Westfalen, Germany). To avoid under-sampling, a post-hoc sample size calculation was performed using G*Power 3.1 software [26]. After completion of the study, a post-hoc power analysis was conducted on the GRF of the main parameters to estimate accurately whether further analysis at trial completion would maintain a sufficient statistical power (80%) and effect size (alpha 0.05) for all the variables, given the number of subjects included. The post-hoc sample size calculations suggested that eight participants were required to detect a moderate difference effect size of >0.5 and a statistical power of >80% between the GRFv for the N and MTP conditions (effect size = 1.25; power = 0.83).

### 2.6. Statistical Analyses

All statistical analyses were conducted using an Excel statistical software package (Ekuseru-Toukei 2016; Social Survey Research Information Co., Ltd., Tokyo, Japan). The assumptions of linearity, normality, and equality of variances were examined, using skewness in statistics and histograms. A skewness of <1 was considered satisfactory. The data are presented as box and whisker plots.

The Wilcoxon signed-rank test was used to compare the mean values of the N and MTP conditions. The level of statistical significance was set at *p* < 0.05, and the magnitude of the difference was assessed by effect size, where the difference was considered to be either small (d: 0.2−0.5), moderate (d: 0.5−0.8), or large (d: >0.8) [27].

## 3. Results

**Figure 3:**
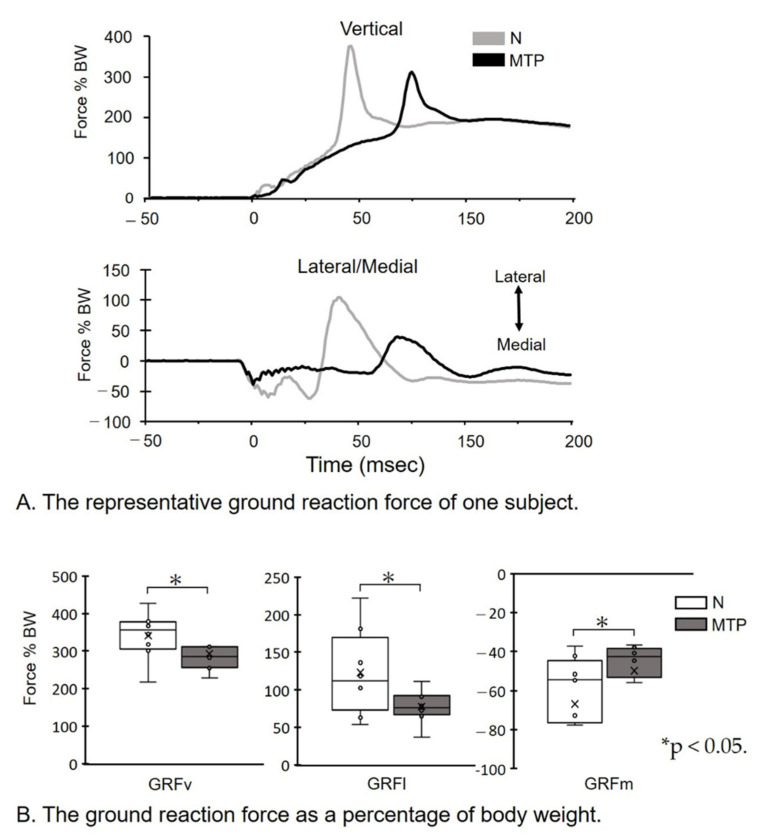
The ground reaction force.

Figure 3 shows the GRFv, GRFl, and GRFm in the N and MTP conditions. The specimen records of GRF amplitude (Figure 3A) were reduced in the MTP condition, compared to the N condition. There was a significant decrease in the GRFv (*p* = 0.017; effect size, d = 0.74), GRFl (*p* = 0.017; effect size, d = 1.07), and GRFm (*p* = 0.017; effect size, d = 0.62) in the MTP condition, compared to the N condition (Figure 3B).

**Figure 4:**
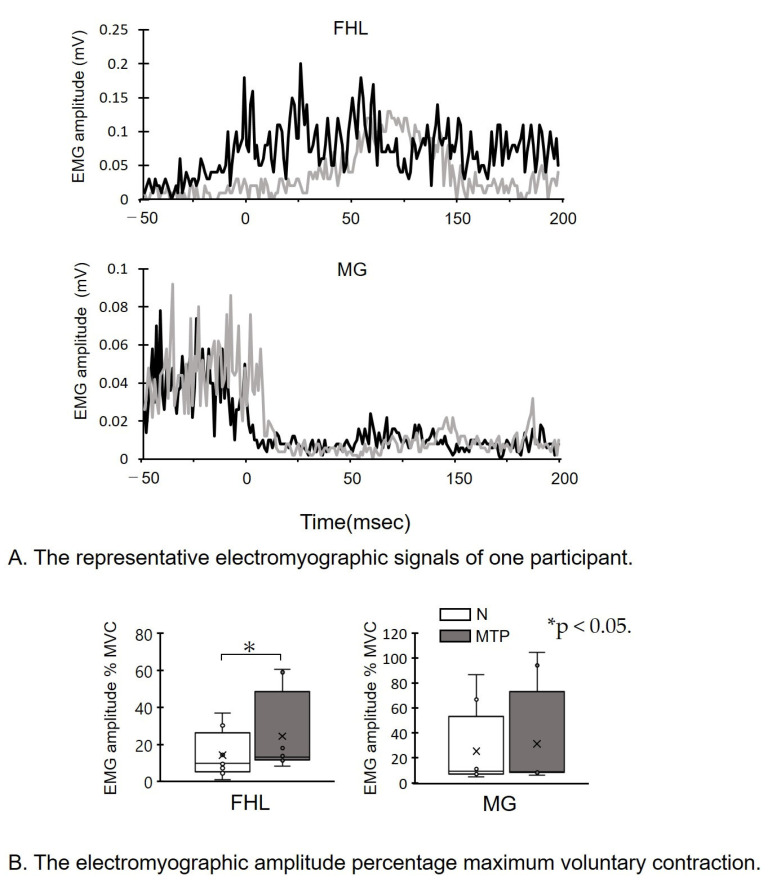
The surface electromyographic signals.

Figure 4 shows the sEMG of the FHL and MG. The specimen records of sEMG amplitude increased in the MTP condition compared to the N condition (Figure 4A). Meanwhile, the FHL activity was found to be dissociated from the MG activity during landing (Figure 4A). There was a significant increase in FHL amplitude % MVC (*p* = 0.017; effect size, d = 0.57) in the MTP condition, compared to the N condition (Figure 4B). There were no significant differences in the MG amplitude % MVC in the MTP condition (*p* = 0.2, effect size: d = 0.16) compared to the N condition (Figure 4B).

The ground reaction force (GRF) during the natural (N) condition (grey) and the metatarsophalangeal joint flexed (MTP) condition (black) are shown for the GRF of vertical force (GRFv), lateral force GRF (GRFl), and medial force GRF (GRFm)

(A) The representative GRF of one subject. GRF data during the 50 ms before and 100 ms following the initial ground contact at 0 ms are shown. Note that the GRF amplitude in the MTP condition is decreased, compared to the N condition.
(B) The GRF as a percentage of body weight (BW). Magnitudes of peak GRFv, GRFl, and GRFm forces as a percentage of BW were identified for each jump, and individual and group means were subsequently calculated. * *p* < 0.05. Lines represent the range of the minimum and maximum. Boxes represent the lower, median, and upper quartiles. The GRF as a percentage of BW in the MTP condition was decreased compared to the N condition.

The surface electromyographic signal (sEMG) of the flexor hallucis longus (FHL) and medial gastrocnemius (MG) during the natural (N) condition (grey) and the metatarsophalangeal joint flexed (MTP) condition (black) are shown.

(A) The representative electromyographic signals of one participant. sEMG data during the 50 ms before and 100 ms following initial ground contact at 0 msec are shown. Note that the FHL amplitude in the MTP condition is increased compared to the N condition.
(B) The electromyographic amplitude percentage maximum voluntary contraction (MVC). sEMG amplitude of FHL and MG percentage MVC. * *p* < 0.05. Lines represent the range of the minimum and maximum. Boxes represent the lower, median, and upper quartiles. The sEMG of FHL in the MTP condition was increased compared to the N condition.

## 4. Discussion

The objective of this study was to investigate the effect of a flexed MTP joint on the landing GRF. The instruction to “flex the MTP joint when you land” reduced the peak GRF compared to the natural condition. The FHL sEMG was increased in the flexed condition compared to the natural condition, indicating that participants were able to increase MTP flexion. Our study is the first to test a modification at the MTP joint to reduce GRF. The results of this study support the hypothesis that increasing FHL muscle activity during landing decreases the peak GRF. The ability to control and adequately absorb GRFs during landing may be the key to preventing injury.

In our study, we found that the FHL sEMG increased with flexion of the MTP joint during landing. The FHL produces higher forces that contribute to toe flexion [28,29].

The strengthening of the FHL muscle may help to increase muscular support of the arch, and thus increase the control of foot stiffness [30,31,32]. Recent studies have suggested that the FHL muscle’s active contractile mechanisms may provide a substantial contribution to the regulation of the stiffness of the lateral longitudinal arch [33,34]. Foot stiffness during landing, gained through an increase in FHL activity, may also decrease high-impact forces. 

In our study, GRFv decreased with increased FHL muscle activity. This finding is potentially important, as a decreased GRFv has previously been reported during drop landings [12]. A high-impact force during landing is a risk factor that has been frequently implicated in lower-extremity injuries [35,36,37].

Note that the GRF is not a direct factor of high internal structure loading, as is commonly assumed [8,9]. However, it might be hypothesized that increases in GRF lead to more impact loading accumulation, which then leads to increased loading magnitude or duration in certain bones, muscles, or joints [10,11]. These results may indicate that increasing FHL muscle activity contributes to the maintenance of foot stiffness, and plays a role in controlling the high-impact force by shock absorption during landing.

In addition to a decreased GRFv, our study found that, as a result of the decreased GRFl and GRFm, the mediolateral GRF amplitude (GRFl and GRFm) decreased with flexion of the MTP joint during landing. A recent study suggested that GRFm induced a valgus in the knee that is related to decreased hip muscle strength and knee injuries, including ACL injuries [38]. A subtalar joint axis of rotation that is positioned more laterally during ground contact, in relation to the GRFl, has the potential to generate a greater supination moment of the foot/ankle complex that could result in lateral ligament damage [39,40]. However, the ligamentous support of the subtalar joint is extensive and not well understood [41]. Sports injuries of the joints, muscles, and bones as a result of impact loading accumulation may affect the vast majority of structures in the body and may be only poorly understood by monitoring changes in GRF [42].

The data in the present study were restricted to the immediate effect of GRF during landing, and several methodological limitations of the current study should be acknowledged. Firstly, we were very cautious during electrode placement, and the validity of FHL surface sEMG measurement was evaluated. sEMG from the neighboring muscles was also observed, to dissociate it when comparing activity from the FHL and MG. It was assumed that the cross-talk from the neighboring muscles has a minimal effect on FHL sEMG during landing. FHL muscle activity should be evaluated using real-time ultrasound imaging, fine-wire intramuscular EMG, and sEMG [43]. Secondly, the dynamic task of landing with flexion of the MTP would perhaps alter the relationship between other muscle activity, and we did not assess the effects of MTP muscles (flexor digitorum brevis and flexor digitorum longus), knee, hip, and trunk muscle activity, and joint moments. Thirdly, only landing for healthy participants was assessed, and we did not assess GRF during dynamic balance. Any patients who have sustained lower-extremity injuries may elicit an alternative response. Therefore, further studies, including long-term follow-up function assessment, are warranted to evaluate the long-term benefits of landing with a flexed MTP joint. It remains necessary to study potential changes or improvements in balance during landing.

## 5. Conclusions

The results of this study indicate that simple cues to increase FHL muscle activity decrease the impact force and, therefore, may be clinically beneficial. This could support the hypothesis that flexion of the MTP joint decreases high-impact forces to prevent injury. Further comparative studies are required to determine the clinical validity and outcomes of rehabilitation therapy.
